# Urban, Suburban, and Rural: Adolescents' Use and Preferences for Fitness Promotion Technologies across Communities

**DOI:** 10.1155/2013/324259

**Published:** 2013-04-10

**Authors:** Erika Mikulec, Natalie Goniu, Megan Moreno

**Affiliations:** ^1^Department of Obstetrics and Gynecology, University of Wisconsin School of Medicine and Public Health, 600 Highland Ave, Madison, WI 53792, USA; ^2^Department of Pediatrics, University of Wisconsin School of Medicine and Public Health, USA

## Abstract

*Introduction*. An understanding of adolescents' use of technology across ages and communities could allow for future targeted obesity intervention strategies. *Methods*. Focus groups of adolescents from rural, suburban, and urban cities in three states were conducted. Focus groups were led by a trained facilitator to explore how participants used technologies and whether they applied them for fitness purposes. All focus groups were audio recorded and manually transcribed. Analysis was conducted by three investigators using an iterative process. *Results*. Five focus groups included adolescents between the ages of 12 and 18 years (20 females and 8 males.) Three themes were derived from our data. First, we found age differences regarding technology applied to fitness. Younger participants described technology as a complement to fitness; older participants viewed technology as a motivator for fitness. Second, differences in fitness approaches existed between rural and urban adolescents. Adolescents in rural communities reported focusing on the outdoors for fitness, while urban adolescents relied on fitness-oriented video games. Both rural and urban teens related having a lack of fitness-focused resources in their communities. *Conclusions*. Our findings indicate differences in adolescents' application of technology for fitness. Despite adolescents' differing uses of technology across communities, a common need exists to expand their resources.

## 1. Introduction

The increasing prevalence of child and adolescent obesity is a significant public health concern [1, 2]. Approximately 34% of the population between the ages of 12 and 19 years is at risk of overweight and 17% is currently overweight [3]. Additionally, 12.6% of adolescents between 12 and 19 years were considered obese [4]. Children who are overweight or obese are more likely to stay overweight or obese into adulthood, thereby increasing their risk of insulin resistance, diabetes, hypertension, and cardiovascular disease [5]. Despite significant efforts towards understanding and intervening in children and adolescents' health behaviors surrounding healthy eating and exercise, obesity remains a significant public health issue. Innovative strategies towards reducing this epidemic are clearly needed.

One innovative approach may involve technology. The majority of adolescents are increasingly “connected” in a virtual world, using different technologies on a daily basis. Adolescents are avid Internet users; over 90% report access and most report daily use [6]. Of these Internet-using teens, approximately half use online social networking Web sites (SNS) such as MySpace (http://www.MySpace.com/) and Facebook (http://www.Facebook.com/) [7]. Other technologies popular among teens include cellular phone, text messaging, and video gaming [8].

Previous research suggests technology may offer great potential for promoting and supporting fitness among adolescents [9]. Communication technologies have been shown to educate via video podcasts about health benefits of exercise and nutrition [10]. Technology may also motivate by inquiring via text message about current activities and nutrition choices, or by sending reminder text pages about a planned activity [11–13]. Finally, technology such as social networking sites provides opportunities to establish and encourage peer-based relationships to share photos or messages. Electronic communication interventions may provide new ways for promoting healthy behaviors. Adolescence is a key period of development during which individuals begin to develop and take responsibility for their own health priorities and behaviors. This may be an appropriate time for the introduction of fitness promotion.

Before such interventions can take place, adolescents' current use and preference in technology must be determined. It is important to recognize that differences in preferences may exist between gender, age, and types of communities and these in turn may influence the effectiveness of technology interventions for health promotion. Understanding these differences may provide insights into targeting appropriate technologies to different adolescent groups towards promoting and supporting fitness. It may further enhance interdisciplinary approaches towards fitness promotion by identifying key roles that are important in different communities.

The primary purpose of this study was to investigate adolescents' preferences for current and future technology use related to fitness. Second, we aim to explore whether differences exist across age or communities with respect to types of technology used and for what fitness purposes.

## 2. Methods

This study was conducted between March 2011 and December 2011 and received IRB approval from the University of Wisconsin.

### 2.1. Setting and Subjects

Focus groups were conducted using purposeful sampling with adolescents from urban, suburban, and rural communities across Wisconsin, Washington, and Ohio. Urban communities were defined as those with a population greater than 50,000, suburban communities had populations between 20,000 and less than 50,000, and rural communities had populations below 20,000. Inclusion criteria for this study were being between the ages of 12 and 18 years and residence in the identified community.

### 2.2. Data Collection and Recruitment

Potential participants were recruited by word-of-mouth recruitment in the different community settings. An adult who had established connections with groups of youth was identified as a leader in each community and was asked to recruit colleagues and friends for participation. Adolescents were able to participate in a focus group after explanation of the study was provided and appropriate consent was obtained from parents and assent from teens. Focus group participants received a $5 gift card as compensation.

### 2.3. Data Sources and Variables

All study data was collected during focus groups. Demographic data including gender, age, and school grade were voice recorded. Participants were asked both open-ended and prompting questions to elicit ideas and conversation among the group. Topics that were covered included definitions of fitness, activities which promote fitness, technology usage, current uses of technology for fitness purposes, school and community resources available for fitness promotion, and future preferences for personal and community technology for fitness improvement.

### 2.4. Analysis

All focus groups were audio recorded and manually transcribed. Three investigators analyzed the transcripts using an iterative process. The investigators initially reviewed transcripts individually and then jointly discussed findings to determine representative themes and quotations using the Constant Comparative Method [14].

## 3. Results

### 3.1. Subjects

A total of 28 teens (20 females and 8 males) participated in five focus groups consisting of between 3 and 8 participants. The average age was 14.9 years (SD = 1.92) and participants ranged from 6th to 12th grade in school. All participants reported using technology on a daily basis. A total of three themes were identified in our data, including differences in use of technology across age groups, the way in which adolescents achieved fitness in different community settings, and similarities in the types of technologies used and amount of resources between rural and urban cities.

First, younger participants described technology as a complement to fitness, *“we're just running and there's nothing to listen to…but when there is music on, everyone feels like oh this is fun, let's run around, let's be fun.”* In contrast, older participants designated technology as a motivator for fitness, *“I mean if I see someone's (social networking website) status is like “going running” I'm like wow, I should probably go do that too.”* Another example quote was *“Whenever you guys say ‘running like ten miles' I'm like maybe I should.”* A final example quote was, “*Yeah like my sister she always puts up like things (on a SNS) for gymnastics like she got a new skill it's like oh I wanna do a new skill now. It really motivates me.”*

Second, differences in fitness activities varied by community location. Adolescents in rural settings reported consistently using outdoor resources for fitness, *“I walk around our property, walk my dog, and just shoot basketball.”* On the other hand, adolescents living in urban areas more often took advantage of indoor fitness options, *“I've got workout videos, like DVD's…and I go to the gym.”* Though differences existed between communities, adolescents across all communities reported exposure to and enjoyment of video game systems that involve physical movement in order to play*; “I got the Kinect and that's definitely for fitness ‘cause everything you do is moving' like your shoulders and stuff…it really gets you moving cause you really get into it.” *Interestingly, adolescents from suburban communities' comments reflected similarities to both urban and rural adolescents' comments.

Lastly, adolescents from urban, suburban, and rural communities related a general lack of fitness-focused resources to their neighborhoods*, “At my house I can't do much, I mean it is two and a half acres, but it's I mean it's not like a big trail or anything to run on and it doesn't have any pavement so I can't do skateboarding or nothing.” *Additionally, *“I wish our school or community had a rec center, with workout machines, a track, and pool, stuff that would like help you, like vending machines with healthy stuff, instead of stuff like chips or candy.”*

## 4. Discussion

Our study demonstrates that adolescents' application of technology differs by age as well as across communities. Younger teens are more likely to use technology in addition to their fitness activities while older teens perceived that they require it as a motivator to accomplish their fitness goals. Adolescents in rural communities utilize outdoor resource; urban teens use those indoors. Additionally, teens regularly use fitness promoting video games and all agree that a common need exists to expand community resources. Therefore, our findings suggest the need for new technology interventions that are targeted to address specific adolescent needs across ages and communities. 

Adolescents' progression through normal stages of development may explain some of the differences in technology use between younger and older teens. Three distinct phases exist in adolescent development: early, middle, and late, during which changes occur in peer interaction and cognition [15]. Early adolescents continue to interact more like children and possess concrete cognition, these attributes may allow them to achieve fitness through playing with technology as a complement to their activities. In contrast, middle to late adolescents become increasingly influenced by their peers' behavior and develop abstract thought processes. These changes may contribute to older teens' perceived need for organized sports or a peer-based motivator for fitness promotion. 

Deficiencies in proximity, availability, and safety of resources for physical activity may explain the differences in fitness resources between rural and urban adolescents in our study. First, proximity to outdoor resources which promote fitness in rural communities is more optimal compared to urban communities where parks and green spaces are less accessible [16]. Second, there are less organized and affordable outdoor activities accessible to urban adolescents, including after-school programs and community centers [17–19]. Lastly, perceived safety concerns by teens in urban communities may impact willingness to engage in outdoor physical activity [20, 21]. 

Despite these differences between ages and communities in their perceived preferences for fitness as well as resources available, a common theme was a desire for additional fitness resources. Given that the vast majority of adolescents have daily access to technology which may include video games, the Internet, and Cell phones, opportunities exist to direct these technologies toward fitness promotion. The findings of our study indicate that a one-size-fits-all approach of providing similar resources to target every age or every community being not likely to be effective. Future technology interventions could target the needs of early adolescents and different communities by providing playful, interactive tools such as implementing video games like Kinect or Wii as part of organized gym or after-school activities. For middle-late adolescents, who prefer independence in activities but require the motivation of their peers, an online SNS group could be supported by schools and community programs which would allow for correspondence about and positive reinforcement of fitness goals. Additionally, technology such as a GPS linked to a phone or computer can provide support and encourage teens to meet and set new physical goals. These options provide ways in which technology can be incorporated to provide accessible, affordable, and safe resources to accommodate the needs of adolescents of diverse communities and across ages. 

Several limitations exist in our study. First, given our qualitative study approach and concomitant small sample size, we cannot ensure that the sample is generalizable to the larger population. Second, we had a greater number of participants who were female compared to male. Further research is needed to determine if needs and preferences are consistent with our findings across other ages and communities. 

Despite our study's limitations, important implications for future technology use to promote fitness exist from our research. Findings may be particularly useful across the interdisciplinary leaders in communities involved in fitness promotion. For example, health teachers for younger teens may wish to suggest ways to incorporate Wii into exercise routines for urban students. High school administrators may wish to consider using social media to promote fitness events or competitions. Nutritionists may consider including discussion of social media advertisements for fad diets in their educational materials. Future research may wish to consider incorporation of social media prompts within interventions or program planning. Given the ubiquitous access to technology by teens and adults alike, our study highlights a few ways in which interdisciplinary team members can consider to provide targeted and relevant fitness promotion to today's teens.

## 5. Conclusion

We found that different adolescent age groups have varied needs and preferences relating to fitness achievement, and because of accessibility, different locations also have differing needs for resources. However, in the end all adolescents describe a greater need for community based resources which can be supported by interdisciplinary teams. Technology presents new opportunities to provide these resources in a way that is tailored to the ages and community of the adolescent group of interest. 
